# Development of Narratives and Belief in a Just World in Victims of Bullying Due to Sexual and Gender Diversity Issues

**DOI:** 10.3390/ijerph191610186

**Published:** 2022-08-17

**Authors:** Adrián Sánchez Sibony, Liliana Jacott Jiménez

**Affiliations:** Department of Developmental and Educational Psychology, Faculty of Teacher Training and Education, Autonomous University of Madrid, 28049 Madrid, Spain

**Keywords:** bullying, sexual and gender diversity, social justice, belief in a just world

## Abstract

Students belonging to a sexual and gender minority go through experiences of injustice in their educational centers and are victims of school bullying. This research analyzes the relationship between these experiences and their influence on the development of the Belief in a Just World, as well as the impact of their experiences on the development of their narratives. Participants are students who have suffered from bullying due to sexual and gender diversity issues during their primary and secondary education stages (ages 15–40 years). Starting from a constructivist qualitative methodological approach, a semi-structured interview was developed as an instrument for collecting data on these aspects. The information extracted was contrasted with the results of the Personal and General Belief in a Just Word Scales. The results of the first interviews provide us with prior information on current identities and narratives and their representations of justice.

## 1. Introduction

For more than two decades, attention to bullying and discrimination based on sexual and gender diversity (hereinafter, SGD) has been held in special importance by researchers and different public entities [1,2,3,4,5]. Specifically, there has been an exponential increase in research in the last decade about these topics [6], as well as a concern that goes beyond the educational context and expands the political and moral framework [7]. Despite the social and investigative impact of bullying due to SGD issues, there is no consensus regarding the definition of its concept [8]. For this research, bullying for SGD issues is understood as a type of gender-based bullying that is identified by denigrating comments or insults and that entails verbal, physical and digital violence toward students who do not follow gender norms and stereotypes, or who have a sexual orientation other than heterosexuality, as a consequence of an internalized and culturally constructed social prejudice [2,9,10,11,12].

Since then, numerous studies of this phenomenon have focused on the different forms of violence and injustice that it entails [13,14,15] and the medium and long-term consequences for the victim [11,16]. In line with this, Poteat, Berger and Dantas [17] indicate that victimization by bullying due to SGD issues promotes a feeling of insecurity regarding the school climate and absenteeism. In a systematic review, Moyano and Sánchez-Fuentes [6] conclude that 29.5% of the investigations affirmed that heterosexual students are less vulnerable to being harassed than students belonging to a sexual and gender minority (hereinafter, SGM) [18]. Verbal aggression is the most frequent, with 70.1% of students belonging to an SGM admitting to having been victims of verbal aggression due to their sexual orientation, followed by physical aggression, suffered by 36.7% of students of an SGM in the US [14]. The results of research in this area indicate that the consequences of exposure to this type of bullying can be poor academic performance, high levels of mental-health problems, such as anxiety, depression and minority stress [19,20], suicidal thoughts or attempts, and risk of substance abuse [8,21].

Meyer and Stader [22] argue that schools are aggressive and hostile environments for SGMs, and specifically for trans youth. The result of this situation for trans youth is exposure to educational inequalities that limit the ability to develop educational resources, access to safe spaces, and support from their peers [23]. In their research, ref. [24] Hong and Garbarino highlight that a highly positive climate for SGM students is promoted in those educational centers with a greater number of students belonging to different minorities (racial and ethnic) and where the teaching staff supports and protects these groups, which reinforces well-being and improves academic performance, as is also the case for trans students [25]. Despite this, trans students are at greater risk of suffering violence in the educational context. In a Finnish study [26] on violence and the school environment, it was shown that 47% of trans students who participated in the study suffered physical aggression, compared to 44% of non-heterosexual students. In addition, Orue, Larrucea-Iruretagoyena and Calvete [27] indicate in the results of their research that boys have higher scores for transphobic attitudes than girls. Other recent studies indicate that intersectionality is a key factor in SGD bullying. SGMs of a different race or ethnicity than the dominant group report higher levels of discrimination compared to their peers within SGD. In addition, this double minority has low levels of social well-being and worse mental health [28,29,30,31]. For example, in the US, white and Latino adolescents present higher levels of victimization than those who are black and Asian, since, in the latter cases, racial discrimination takes on a more relevant role and allows the development of tools to face discrimination based on SGD issues [32,33].

Finally, a new trend in research tries to delve into the social and environmental factors that are part of situations of unjustified violence and bullying due to SGD issues [13].

## 2. Second-Order Theories

Fenaughty [34] calls this trend second-order theories, since they move away from the first order, focused on the profile of the victim and the aggressor, and focus on the mechanisms of social oppression of some identities through mechanisms of unjustified violence in the educational context. This type of research is developed from a social justice perspective and focuses on human development, in addition to analyzing the school climate [17], well-being and mental health [29], resources, recognition and teacher training [35], protective and preventive factors [21] and the development of the personal narratives of the victims [36]. Poteat, Mereish, DiGiovanni and Koenig [37] stress the importance of developing specific policies that address the problem of bullying due to SGD from a more structural and systemic approach that takes into account the different social contexts in which it occurs. Therefore, from our perspective, what these authors are proposing is to adopt an approach that could be considered closer to a social justice approach, although they do not define or use this term. Other authors, such as [38] Gegenfurtner and Gebhardt, maintain that this type of bullying is also a form of social oppression toward SGMs. It is on this last current of knowledge that our research is framed.

### 2.1. Social Justice and SGD at Schools

Fraïssé and Barrientos [39] consider the term homophobia from a psychosocial approach as “a complex system, in which heterosexism, sexual prejudice, heteronormativity, sexism and male dominance interact with one another” (p. 9). For our research, we avoided the use of the term homophobia, due to the pathological meaning of the term and the invisibility of other realities within SGD [40,41,42]. In the educational context, bullying for SGD issues is considered an international problem of social injustice that affects all students equally [43]. Some authors have tried to address this problem from the perspective proposed by Freire [44] on oppressive pedagogies and the literacy of oppressed people. For example, Fenaughty [34], in his queer literacy project, explains how he developed specific content following the UNESCO recommendations [45] on how to address situations of unjustified violence and bullying due to SGD issues. In the last decade, scientific interest has arisen in the application of queer literacy to intervene in the forms of oppression toward SGMs exerted by heteropatriarchal social and political norms [46,47,48]. Considering this problem as a form or system of injustice that occurs in educational centers, our research moves away from the queer literacy model and is based on the social justice model proposed by Nancy Fraser [35,49,50,51,52,53]. According to the author [54], “the most general meaning of justice is parity of participation” (p. 39). This situation of justice starts when the system applies specific requirements, such as the redistribution of resources, social and cultural recognition within organizations and political representation (model of the triple dimension of social justice), that is, the possibility to give people who belong to a collective within an organization voice and power in decision making. An example of the application of the three dimensions in the educational context is provided by Belavi and Murillo [49], redefined as Redistribution of opportunities and benefits of education, Recognition of cultural values and social diversity and School governance (in addition to two dimensions that address democracy and education, Democratic School Culture and Critical and Participatory Curriculum).

### 2.2. Climate, Protective and Preventive Factors and Well-Being

Montero and Cervelló [55] define the school climate as a “multidimensional construct that embraces different definitions and critical characteristics” (p. 138), under a series of standards, objectives, interpersonal relationships, educational practices and organizational structures, to establish a positive environment that supports people emotionally and physically. Grounded in the imaginary of SGD, Marchueta and Etxeberria [19] affirm that a school climate that promotes social justice and well-being for sexual minorities must contemplate anti-discrimination and anti-harassment policies, promote awareness and training of teachers and staff of the center, that its curriculum integrates content on diversity and sexual and gender development and provide resources as sources of information and advice for this minority within the student body. Espelage et al. [2], in a review of the scientific literature on prevention and protection factors for students belonging to an SGM, highlight that a positive climate toward GFD reduces the negative consequences of this type of bullying, in addition to improving school participation of the collective.

In contrast, Frost et al. [29] show how, despite achieving a more positive climate toward GFD, young SGMs continue to experience minority stress and engage in stigma-focused cultural discourses [56]. In addition, violent and discriminatory behaviors due to GFD are reinforced over time between peers and end up being established within the group [57]. A negative school climate for SGMs encourages the use of intolerant and pejorative language, as well as relationships of subordination and domination, intimidation and harassment [58].

Despite the above, Hong and Garbarino [24] analyzed the scientific literature from a socio-ecological perspective [59] around this problem that affects students, their families, teachers and other educational personnel. The authors concluded that there are positive advances during the first decade of the XXI century on the issues of social justice that affect SGMs and that such social advances have a positive impact on the well-being of the student body belonging to SGMs.

#### Gay–Straight Alliances

Parker and Bickmore [60] suggest that creating safe and open spaces for critical communication and discussion of sexual and gender identities not only allows students to share their experiences and perspectives, but also to broaden knowledge and build fairer thoughts in matters of high moral sensitivity. An example is the clubs or Gay–Straight Alliances (GSA) in US high schools. According to Mayo [61], the GSA are student-led clubs that promote support and improvement of the school climate for SGMs through activities and reflection on social norms around SGD. The development and configuration of these school groups led by a tutor have opened a new line of research between the scientific society on their benefits for students and the school climate [62]. The presence of GSA has shown benefits in the well-being of students belonging to an SGM and the promotion of a positive school climate for this type of student [63,64]. It also positively influences the empowerment of young people belonging to an SGM and their participation in social justice issues [65,66]. Despite promoting these positive factors for the school and the SGM student body, the GSA are also the center of criticism from the student body or the staff of the school; they suffer bureaucratic obstacles to carrying out their activities or they do not have the support from all families due to social oppression and injustices suffered due to belonging to an SGM [67,68]. They are also considered promoters of traditional sexual identities, such as heterosexual, homosexual and bisexual labels, giving less visibility to the new identities of young people belonging to SGMs [69].

### 2.3. Identity Development and Narratives

During adolescence, young people develop their identity, that is, the awareness of who they are and the understanding of their personality [69]. Savin-Williams [70] indicates that the identities of young people belonging to SGMs are being redefined in the last decade, questioning the labels assigned at the end of the XX century (homosexual, heterosexual and bisexual). Furthermore, it has been shown that sexual and gender identities can fluctuate throughout life [69]. In line with the above, several investigations affirm that allocating resources and allowing safe spaces for SGMs within educational centers has great, positive repercussions on the personal development of students [63,71].

The narrative is the process of giving meaning to lived experiences through the development of an individual discourse [72,73,74]. The configuration of identities and personal narratives of youth belonging to an SGM is marked not only by the experiences lived, but also by the social and political context in which they are located and the relationship between subjective meanings that arise in the course of their lives. In addition, the development of sexual and gender identity can have different discourses throughout life that, on some occasions, become contrary to one other; that is, each person has their own identity configuration process [75], at least as far as homosexual men in current American culture are concerned.

Cohler and Hammack [73] identify the construction of two main master narratives in the development of the identity of homosexual people within American culture: the narrative of struggle and success and the narrative of emancipation. Although these narratives reflect different historical moments in the cultural construction of homosexuality, both are still present as master narratives of the identity of SGMs today, but both narratives do not understand all the diversity of experiences and narrative discourses of the youth of SGMs [76].

#### 2.3.1. Narrative of Struggle and Success

This master narrative unfolded in SGMs born in the 1960s and 1970s, who came of age in the 1980s and 1990s. This period, in which the struggles for the rights of homosexual people began, exposed people to social experiences and discourses that had not been considered before [77]. It arose as assumption and integration of stereotypes and negative cognitions associated with sexual minorities. It is identified by the fight against the injustices suffered by the collective, with an activist alignment that rebels against the stigma it suffers, and, ultimately, with a narrative empowered by success in managing these injustices of heterosexist origin. This stigma was enhanced by minority stress and led to substance abuse, suicide attempts or ideas, and mental illnesses such as anxiety, stress and depression as a result of their struggle. Therefore, its focus is also centered on the active struggle and resilience against the heterosexist world. Present in this narrative is the need for recognition and social acceptance through changes in the environment, professional assistance and organizations that support the personal development of SGMs. This narrative depends on the discriminatory discourse and the silenced voice of SGMs that prevails in the dominant heteronormative culture.

#### 2.3.2. Narrative of Emancipation

The narrative of emancipation takes place during the most recent decades (the late 90s and early 21st century) and is characterized by not identifying with the stereotypes linked to SGMs. It also distances itself from the association of suicidal ideas or attempts as a result of oppression exerted by the dominant norm for GFD issues [78]. It does not defend gender identity and rejects the use of labels associated with the collective. Its ideology is more associated with breaking down the idea of binary gender (queer) and usually appears in SGMs that belong to larger urban areas and have a greater socio-economic level, so they have more access to resources that allow them to lead changes and make speeches. The main objectives of the people who identify with this narrative are aimed at improving their quality of life to a point where GFD issues do not predominate. In other words, in the emancipation narrative of sexual and gender identity is not the basis for identity development. For the authors [73], formulating a narrative whose main concerns are the achievement of personal goals and the improvement of the quality of life that is emancipated from the stigma suffered by SGMs, and integration of the heteronormative life course is manifested. On the contrary, from a social justice perspective, this argument would be refuted when considering the parity of participation of the dominant heteronormative and heteropatriarchal social group, understood as a situation of justice that SGMs with emancipatory narratives try to reach.

Currently, youth belonging to an SGM live with both narratives simultaneously [70], so this influences the development of their identities [73].

It is necessary to highlight that the bibliography that appears in this section refers to studies whose main sample is homosexual and bisexual people, more specifically, on homosexual men, moving away from the development of the identity of transsexual, transgender or intersex people.

### 2.4. Belief in a Just World

People need to believe that the world is a just place, where everybody gets what they deserve in a meritocratic way; that is, it is the belief that people get rewards or punishments for their actions, moving away from the concept or reality of social injustice. In this manner, BJW is meaningful for people, since it allows them to have a sense of control over their environment via their previous actions [79,80]. If they do not believe it, they assume that the disagreeable situations that happen to other groups of people can also happen to them, leading to a feeling of anxiety or lack of control over the situation [81,82]. In response to this suffering, they tend to blame the victims of injustice for their situations. Thus, BJW justifies and perpetuates an unequal economic and social system, supporting authoritarian positions and discriminatory attitudes toward disadvantaged or underprivileged social groups [81,83].

In the educational context, the Just World Belief (JWB) exerts a great influence on the different roles that interact within the educational community. Albalá, Etchezahar and Maldonado Rico [84] evaluated the relationship between the orientation toward inclusion and the BJW level of 476 students completing Teaching Degrees in Early Childhood and Primary Education at the Autonomous University of Madrid. Their results confirm that students with a low BJW have an indirect relationship with an orientation toward educational inclusion. On the contrary, a high BJW in the student body is related to a low orientation toward educational inclusion due to their interpretation of injustices. As a noteworthy fact, future teachers with a high BJW defended the argument of relocating students with special educational needs to educational centers with specialized care.

#### Belief in a Just World and Bullying

In recent years, different interesting studies have emerged regarding the relationship between BJW and the experiences of victimization and injustice lived by young people [85]. In China, in a sample of 750 students aged 10 to 16, it was concluded that a high BJW was indirectly related to low participation in school cyberbullying situations, since the world was considered a fair place, actions were not interpreted by other people as aggressive. At the same time, a high level of family attachment is related to a high BJW in understanding the world as a safe place [86]. Similar data were reported in a western context, specifically in 187 Portuguese students [87]. Donat, Umlauft, Dalbert and Kamble [88] analyzed and confirmed the mediating effect of the evaluation of fairness in teachers by students and the relationship with bullying behaviors. When teachers’ acts are perceived as fair individually, bullying behaviors are lower.

For Donat et al. [85], the BJW is an important resource for victims of bullying that allows them to overcome the injustices, obstacles and conflicts present in their lives. Students with a high BJW feel less victimized and consider that the treatment they receive is fair both from their classmates and from teachers, as well as considering that the victims of bullying deserve to be in that situation because of their actions. Currently, Barreiro, Etchezahar and Prado-Gascó [89] affirm the differentiation of two dimensions in the BJW construct, the personal (BJWP) and the general (BJWG). The BJWP is situated in the belief of a fair world in personal events throughout life, in addition to considering the acts themselves as fair. The BJWG is situated at the level of the injustices that affect other people and is usually situated at lower levels than the BJWP.

With all the information presented above, we start from the hypothesis that the situations and experiences of injustice and bullying toward SGMs in educational contexts influence the development of their narratives and sexual and gender identities. Likewise, we consider that there is a relationship between the BJW and the experiences of victimization and injustice lived. We intend to achieve three main objectives:

1. Analyze the personal narratives of young people that have experienced bullying at school due to SGD issues with respect to the developmental trajectories of their sexual and gender identities.
2. Explore the relationship between the experiences of harassment and injustices experienced by students belonging to SGMs in the educational context from the development of their narratives.
3. Know their Belief in a Just World (personal and global) and to explore the relationship between these beliefs and their narratives.

## 3. Method

### 3.1. Participants

A non-probabilistic accidental sample was used to carry out this research. A snowball dynamic was also used to enlarge the sample. We intended to reach a population composed of people who have been victims of bullying for SGD issues during the stages of Primary Education and Compulsory Secondary Education; that is, we expected to collect experiences of all kinds of realities within the imaginary of SGD (homosexuality, heterosexuality, bisexuality, people who identify as trans, non-binary gender or gender fluid). The research is currently in the data collection and analysis phase, but the experiences of 15 young participants who have experienced SGD bullying have already been analyzed. The sociodemographic data of the sample collected through an ad hoc questionnaire are presented below (see Table 1 and Table 2). As can be seen, the main results obtained are largely from homosexual men.

Of the 15 participants, three self-identify as women and 13 self-identify as men in terms of their biological sex. Regarding sexual identity, only one participant identified as gender fluid, as opposed to gender identity, where two persons identified as gender non-binary and one person identified as queer. Regarding sexual orientation, five persons identified as bisexual, nine as homosexual, one pansexual and one as queer. Only two respondents confirmed belonging to the Christian religion, while the rest considered themselves to be atheists or flowed between atheism or agnosticism. As for the population where they were victims of bullying, nine persons belonged to rural populations or localities with an intermediate population density and six individuals belonged to large or small urban areas.

### 3.2. Procedure

Following [90] Massot, Dorio and Sabariego, we chose a qualitative approach to explore the discourse of the sample, while giving voice to their experiences and opinions through their narratives. In our research, we follow the model of Frost et al. [91], which starts from an integral methodological approach in which different constructivist and pragmatic perspectives converge. From a constructivist approach, the focus is on the personal narratives and experiences lived by the participants about the subject under study, and where the processes of construction of the meaning of the subjective experiences narrated are analyzed. From a pragmatic approach, some of the topics are addressed, as well as specific questions about the content of the phenomena under study.

To explore the discourse of the sample, the semi-structured interview was chosen as the main information-gathering tool [92], as it is a practice in which meanings are made and knowledge is generated based on lived experience, drawing social change through the subjective constructions of the participants. In other words, life experiences as a whole can be analyzed in relation to larger master narratives or cultural narratives [93].

The process of developing the interview began by seeking or developing questions or subgroups of questions framed in four specific issues, identity, bullying experiences, the climate and school characteristics and representations of justice. The objective of each specific issue is to deepen the areas of knowledge on which the theoretical framework of this research is based. The questions required to be flexible enough to provide the maximal information from the sample, reflecting the processes of construction of personal narratives, as well as contents of the description of occurred events or peer and family relationships, that is, both from the personal perspective, as well as the situation of the environment [91].

The following table (Table 3) shows the relationship between each specific issue of the interview questions and the areas of knowledge and research in which the information collected is explored for subsequent analysis:

Following the model of Corbin and Strauss [94], once the initial questions that made up the first version of the interview were defined, it was applied to a pilot sample (four participants). The data were analyzed with the qualitative analysis tool NVIVO12. Afterward, the questions were refined based on the data received and those that did not go deeper or did not provide the desired information were modified. Finally, the information collected on the last specific issue was contrasted with the Belief in a Just World Scale (Lipkus scale) [83]. Once the second interview model was defined, a new pilot sample was used again and its results were contrasted with the General and Personal Belief in a Just World Scale [89]. The objective of using a pilot sample again was to confirm that the changes applied to the questions collected the desired information.

Finally, the interview was sent to an expert group [95] in qualitative research and the areas of knowledge that make up the theoretical framework. This group was composed of four experts from different universities in Spain and Argentina, except for one member who held a doctoral degree and was a Human Rights professional. For the inter-expert judgment, an evaluation rubric designed ad hoc was used to estimate the relevance and clarity of the questions that make up the semi-structured interview. The following table (Table 4) summarizes the profile of each member of the expert team.

The data obtained from the interviews were analyzed with the qualitative analysis tool NVIVO12. For this purpose, the interviews were transcribed, and common themes (nodes) were identified among the data obtained, creating a system of nodes for the construction of relationships and meanings [96]. Currently, the node structure has not been fully developed, as the research has not yet been completed.

## 4. Results

As indicated above, this research is in the data collection and analysis phase. Currently, 15 participants have been interviewed and have also responded to the Personal and General Just World Belief Scale.

### 4.1. Narratives and Life Course Results

Regarding the data obtained on narratives and life course discourses, we found characteristics of both narratives (narrative of “struggle and success” and narrative of “emancipation”). For example, to a varying degree, each participant affirmed being an activist or believing in the movement and struggle for sexual and gender freedom rights. Interview #5 states, “*I consider myself an activist person although I do not belong to any organization. I also support the cause and I think we should be united. I try to go to all possible events*”. The performed activism could range from participating in events and protests, or simply being informed and supporting a speech. This argument is closely linked to generations in the U.S. context, where generations born and educated during the 1970s and 1980s, whose discourse is usually situated in the narrative of struggle and success, were highly exposed to riots, discrimination and unjustified violence toward SGMs. It is not the case in the Spanish context, as age does not seem to be relevant in terms of self-determination as an activist. On the other hand, we did find a discourse similar to the emancipation narrative regarding self-determination of sexual and gender identity that moves away from the use of currently established labels (LGBTTIQ+) in the discourses of those who came from a rural population or with an intermediate population density when compared with those participants from an urban context. As a highlight, there was a dissident argument against the use of labels, either due to rejection or disconformity, for their own identity, while they supported the need for them to exist to make other realities visible. On the other hand, those who came from large or small urban areas and whose age was over 24 showed a degree of appropriation and assumption of the stereotypes socially assigned to homosexuality. People whose age was less than or equal to 24 years, regardless of the type of population from which they came, distanced themselves from or rejected the stereotypes established in society about homosexuality. This finding is in line with other studies suggesting that young millennials and young adults are increasingly openly rejecting the use of traditional labels to account for their own sexual orientations, experiences and identities [97,98].

Thus, we can consider that the first research objective, “*to analyze the personal narratives of young people who have been bullied for SGD issues concerning the developmental trajectories of their sex and gender identities*” has been achieved, despite not having obtained all the data necessary to present conclusive data. More information needs to be gathered from other experiences to define a more consolidated narrative or narratives and their relationship to life discourse.

### 4.2. Bullying Experiences and School Environment Results

Regarding the experiences of bullying reported by the sample, we do not find differences in the educational stages during which the aggressions occurred and the school ownership. From the 15 participants interviewed, 13 reported being bullied due to SGD issues in Compulsory Secondary Education (ESO); eight participants also mentioned that the aggressions started during Primary Education and five participants confirmed that they were bullied during High School. It is necessary to highlight one experience of bullying only during a bachelor’s degree. In this experience, it was indicated that, due to the rural population and religious and private school, in which positive relationships within students were promoted, the students did not develop discriminatory or violent dynamics toward SGMs, because there was a sense of family among the members of the school community in secondary school. Interview #8 states, *“In my class we were a family, we were educated to be a family. Each classmate was different and that didn’t matter, because we all helped each other”*. The aggressions began at the time of the Baccalaureate stage. In two cases, school bullying due to SGD went on for approximately 2 years, whereas the rest of the cases varied from 4 to 10 years. Finally, five participants reported that they not only received verbal aggression, but also threats, personal harassment and physical aggressions.

In all the schools in which situations of bullying due to SGD issues occurred, a negative climate was perceived by the sample. Only in one school were reactive measures taken to address the discrimination toward the SGD and proactive measures in terms of student training with the participation of associations and activist groups. These facts occurred at the end of the 2010s, so it is closer to the present day. In addition, the ownership of the school was public. Regarding the rest of the participants, they did not perceive that the teachers had a specific background in SGD issues. In two interviews, teachers were referred to who had a positive impact on the victim by showing alignment and support; in addition, these teachers were perceived as belonging to SGMs. The sexuality and gender training received was limited to one or two one- or two-hour sessions during Compulsory Secondary School. These sessions were focused on sex education, specifically on the prevention of STDs from a heteronormative perspective. There were also no reported safe places, but they had chosen, for example, the library. In nine interviews, the library was reported as a safe place during breaks because there usually were not any students and it was supervised by a teacher. Furthermore, except in the school where proactive initiatives were taken following the needs of students belonging to an SGM, the experiences reported in the sample considered that the teachers were aware of the violence and injustices suffered by students. Only one experience was reported in which a teacher intervened to stop verbal aggression during class.

Taking into account all of the above, it seems that there is empowerment or an activist sense in those people who have had preferences and support from their families or the school during periods of bullying, while those who have not had these resources or support tend to have a more defeatist or victimizing discourse, as shown in interview #7, who indicates, *“if I had not had to leave my town to avoid this type of situations now I could be a lawyer, as I wanted to at first, and not have studied Vocational Training”*. Thus, we consider that we are reaching the second objective of this research, which is to explore partially the relationship between the experiences of harassment and injustices experienced by students belonging to SGMs in the educational context from the development of their narratives. It is still too early to present further results in this section.

### 4.3. Representations of Justice and BJW Results

All participants recognized they had been treated unfairly when they experienced bullying due to SGD issues. They also considered that the teachers and the school should have intervened when the first aggressions were detected. Interview #9 states, “*they should have stopped it right then and there, not let it happen in class anymore. It was embarrassing and in the end no teacher did anything*”. One argument has been identified in the sample that excuses the teachers for their lack of intervention due to the socio-cultural and political context during the period when bullying occurred. According to this part of the sample, at that time, SGD was a taboo subject and they understand that teachers had no interest or resources to be instructed. In spite of the above, the whole sample qualifies the situations experienced by people who belong to an SGM as unfair. Except for three cases in which the entire society was responsible, the rest of the sample holds the political and legal sphere responsible for making the world an unfair place. Interestingly, the ones whose representations of the situations of injustice and violence experienced were due to SGD issues, were closer to the social justice approach proposed by Nancy Fraser [54]. With regard to knowledge of anti-discrimination laws and protection rights, most of the sample was not familiar with the specific legal framework on SGD issues. Only in four cases was there knowledge of cause on this issue, three of them due to the fact that they were associated with organized activist groups and the other case was due to academic achievements related to this issue. There was also unanimity in the opinion that some sexual realities (heteronormative) were privileged over other realities in schools, and that cissexual or cisgender and heterosexual students had a privileged situation compared to other realities within the schools. On the other hand, the scores obtained on the General and Personal Belief in a Just World Scale (see Table 5) were quite varied in relation to sociodemographic factors, experiences of bullying and unjustified school violence, and identity. Higher PBJW scores are obtained than GBJW scores [89]. In the PBJW, the scores obtained can range from 35 (maximum) to 7 (minimum); whereas, in the GBJW, the scores obtained range from 30 (maximum) to 6 (minimum) points.

With this information, we could indicate that the third objective of the research, “*to know the Belief in a Just World (personal and global) and to explore the relationship between these beliefs and the narratives*”, has also been achieved, but it is necessary to increase the information with more interviews.

## 5. Discussion

It is too early to present conclusive results because this research is still in its data collection and analysis phase. The saturation principle [99] has not been achieved yet, so the data collected in the interviews and the Personal and General Beliefs in a Just World Scale would provide more diversity in the results. However, while we are still compiling and analyzing the data, we can discuss the information collected so far, which is open to further discussion. With respect to the analyzed narratives of our participants, we did not identify completely the two master narratives described in the study conducted in the United States [73]. In our study, there is no alignment in the identity of the people interviewed with the narrative of struggle and success or the narrative of emancipation. Some common characteristics of both have been found in their personal narratives, which may be related to the fact that both narratives coexist together in the same socio-cultural context [70,73]. In terms of sexual and gender identity development, a later development and subsequent self-determination of identity are recurrent in some discourses. A later development of sexual identity because of the great lack of referents, teacher training, educational resources and recognition of the SGD problems that occur to a greater extent in rural populations [100]. The sample that came from rural areas confirmed having become aware of their sexual and gender identity after moving to cities with a greater population and, consequently, with greater diversity. In terms of self-determination of sexual and gender identity, this part of the sample distanced themselves from the socially established labels around SGD, i.e., by not identifying with a specific fixed sexual orientation or gender role, and not using the established labels for it. The latter leads us to assume that they move away from the stigma of identifying themselves as members of the LGBTTIQ+ collective, but they still support the promotion in terms of legal protection and the fight for human rights around SGD. In addition, they consider that they are promoting their own individual type of activism in the different spheres and contexts of their daily lives.

Out of the 15 interviews conducted, the proportion of men versus women in terms of biological sex is higher, with 12 men and 3 women. Although all persons volunteered to be interviewed, we consider some relationship with the results of Orue, Larrucea-Iruretagoyena and Calvete [27] on the proportions of bullying by gender. Males suffer more bullying than females and it is males who perpetrate more of these bullying situations. This last fact also coincides with the bullying experiences of the sample, since it was usually men who promoted such aggression.

Regarding the scores obtained on the General and Personal Belief in a Just World Scale, we found very different scores. On the one hand, the PBJW scores obtained are high in a large part of the sample, indicating that the interviewees consider that they have been and are treated fairly in the different fields of their lives. On four occasions, the PBJW scores obtained were very low in comparison with the average score of the scale (M = 21). These cases coincided with the sample suffering double discrimination due to racial, cultural and physical issues or abuse by people around them. Regarding GBJW, we found scores under the average of the scale (M = 18) related to two factors: the vocation for the field of social progress and those who attended private Christian schools. Two respondents with a below-average GBJW score had attended public schools, but one of them belonged to a non-profit association promoting SGD equality activism and the other worked as a social worker with immigrants. This could result from constant exposure to other marginalized backgrounds, although further interviews with similar profiles would be needed to affirm this idea. Curiously, these two participants also scored below average on their PBJW. Another segment of the sample that also presented below-average scores on their GBJW were those who suffered bullying for SGD issues in private or public-funded Christian schools, but contrary to the two cases described above, these individuals obtained above-average scores on their PBJW. It could be argued that in these types of Christian schools, realities such as global poverty, food shortages and values such as solidarity are taken into consideration, but the idea that there is a God who is fair to people is reinforced, so the PBJW is reinforced. In addition, two of the people who attended concerted and private Christian schools identified themselves with the Christian religion, so they have a high religious orientation. This is in line with the results of previous studies confirming the positive correlation between religious orientations and BJW [89,101].

We can consider the idea that there is a relationship between experiences as a victim of bullying for SGD issues, school ownership and social injustices toward SGMs and the development of a personal narrative and life course discourse. Specifically, the experiences of bullying, the school ownership and the social injustices toward SGMs could be mediators of the configuration of the personal narrative, the discourse of life and BJW, but these results need to be confirmed by future qualitative and quantitative studies that measure the relationship between these factors. We could also consider the idea that the master narratives that predominate in SGMs in the U.S. context, specifically regarding sexual orientation [73,76], do not apply to the Spanish context. A possible new purpose of this research might be the identification of master narratives in the Spanish context. In line with Hong and Garbarino [24], positive improvements have also been identified regarding situations of injustice toward SGMs. The life course discourses of the younger people in the sample deal more openly with their sexual and gender identity and sexual orientation. This may occur due to the emergence of new models and referents through mass media such as the internet and social networks.

Finally, it is necessary to emphasize the wide scope of the theoretical framework supporting this research as a possible further limitation. Defining the relationship between the development of narratives in SGM according to their experiences as victims of bullying due to SGD issues, considering the model of social justice proposed by Fraser [54] in schools [49], their representations of justice and their BJW, may be too ambitious, but it is considered necessary to understand the educational gaps and possible new repercussions. Another limitation is access to a larger sample. It is difficult to find volunteers who are prepared to talk about experiences that may have been traumatic. Not everyone is interested in sharing such experiences. We also consider the context to which the sample belongs, in this case, the Spanish context, a limitation, since it does not represent other realities at the international level. We do not consider the size of the sample as a limitation because this is qualitative research and if we so increased the sample size, we would detract from the validity and value of this approach. In this way, we aim to contribute to the scientific literature on preventive and protective factors toward SGMs, in addition to promoting the use of qualitative methods that focus on experiences and life course discourses as the main sources of information for research on social change and the development of sexual and gender identity [91].

### Social and Practical Implications

A series of suggestions at both the societal and practical levels can be considered following the outcome of this research. In terms of social implications, understanding the cognitive construct of SGD victims of bullying may clarify the failures or gaps that result in late developments of sexual identity self-determination. Moreover, understanding that the world is not a just place implies fostering critical thinking about representations of justice and moving away from authoritarian or meritocratic positions, i.e., promoting equality among people regardless of the segment of the population in which they find themselves or the group with which they identify. These indications derive from approaching the issue of injustices toward SGM from a social justice perspective (second-order research), rather than from the individual consequences that can result from SGD bullying and social injustices.

The practical implications of this research could be situated in the school context. The data from the interviews may help to identify resources to address the needs of SGMs in the school, as well as the necessary cultural awareness within the curriculum and school organization. Finally, another practical implication could be enabling channels of representation and how best to do this democratically [49] based on the experiences of the sample.

Finally, we consider that continuing to address this issue from a critical and social justice perspective is of great importance and necessity to improve public awareness and avoid situations of aggression or murders, such as those that have occurred in recent years, such as the murder of Samuel Liz in the summer of 2021 in Spain [102].

## 6. Conclusions

The social injustices of SGD and its reality in the educational context continue to be a problematic issue that, despite the last two decades of scientific research, have not been solved. What can be confirmed is that there have been significant improvements in this field. The previous results of this research show us the change of perspective in terms of cultural recognition. There are more teaching resources, action plans and awareness programs on SGD available to teachers, students and families, but these are not incorporated into the curriculum yet. On the other hand, younger generations have more opportunities to elevate their voice in schools. An example of this can be found in interview #11, where the participant indicated, “*after the bullying incident, the head teacher asked me and a classmate what we needed in relation to the sexual education workshops organized by the school. We asked to contact associations on sexual diversity and the center organized workshops on this topic*”. This would not have been possible in previous generations. Similarly, we have also observed how the configuration of narratives changes in the homosexual population, but in contrast to the U.S. context, whose changes are constructed according to generation, in the Spanish context, it seems that the difference in narratives is based on the size of the population that the SGMs belong to. As for the BJW, it seems that the results of the previous research cited are confirmed, contributing to the scientific literature in the study of the Spanish population. Once this research has been completed, definitive conclusions can be presented.

## Figures and Tables

**Table 1:** Sexuality and Gender: individuals’ data.

Interview	Age	BS ^1^	SI ^2^	GI ^3^	SO ^4^
**1**	34	Man	Man	Male	Homosexual
**2**	33	Man	Man	Male	Homosexual
**3**	42	Man	Man	Queer	Gay label, but recently changed to Queer.
**4**	32	Woman	Woman	Female	Homosexual
**5**	27	Man	Man	Male	Bisexual
**6**	22	Man	Gender fluid	Non-binary/fluid	Bisexual
**7**	36	Man	Man	Male	Homosexual
**8**	33	Man	Man	Male	Homosexual
**9**	37	Man	Man	Male	Homosexual
**10**	26	Man	Man	Male	Bisexual
**11**	18	Woman	Woman	Non-binary/fluid	Bisexual
**12**	33	Man	Man	Male	Pansexual
**13**	24	Woman	Woman	Female	Bisexual
**14**	36	Man	Man	Male	Homosexual
**15**	24	Man	Man	Male	Homosexual

^1^ BS: Biological sex; ^2^ SI: Sexual identity (self-categorization); ^3^ GI: Gender identity (self-categorization); ^4^ SO: Sexual orientation.

**Table 2:** Sociodemographic data.

Interview	Age	Last Degree Achieved	School Ownership	Center Location	Religion
**1**	34	Degree/Bachelor’s degree/Diploma	Concerted (Christian)	Rural populations	Christian
**2**	33	Degree/Bachelor’s degree/Diploma	Public	Rural populations	Atheism
**3**	42	Postgraduate	Private	Large urban area	Atheism–Agnosticism
**4**	32	Postgraduate	Public	Medium urban area	Atheism
**5**	27	Degree/Bachelor’s degree/Diploma	Concerted (Christian)	Rural populations	Atheism
**6**	22	Vocational training	Public	Large urban area	Atheism
**7**	36	Vocational training	Public	Rural populations	Atheism
**8**	33	Ph.D.	Concerted (Christian)/Private	Small urban area	Christian
**9**	37	Postgraduate	Concerted (Christian)	Rural populations	Atheism
**10**	26	Degree/Bachelor’s degree/Diploma	Private (Christian)	Rural populations	Atheism
**11**	18	High school	Public	Medium urban area	Atheism
**12**	33	Postgraduate	Public	Rural populations	Atheism
**13**	24	Compulsory Secondary Education/EGB	Public	Rural populations	Atheism
**14**	36	Degree/Bachelor’s degree/Diploma	Public	Medium urban area	Atheism
**15**	24	Degree/Bachelor’s degree/Diploma	Private	Medium urban area	Atheism

**Table 3:** List of thematic blocks in the interview and areas of knowledge.

Specific Issues	Areas of Knowledge and Research
Identity	Personal and Master Narratives in SGMs
Bullying experiences	Bullying due to SGD issues
Climate perception and school characteristics	Model of social justice applied at schools
Representation of justice	Representations of (in)justice, violence, oppression, privilege

**Table 4:** Expert team members’ profiles.

Expert Team	Universidad	Specialty/Area or Line of Research
Expert 1	Buenos Aires University	Social Psychology, Social Identity and Prejudice
Expert 2	University of the Balearic Islands	Gender Studies and Gender Violence
Expert 3	Pablo de Olavide University (Seville)	Peer School Violence
Expert 4	Complutense University of Madrid	Sexual Diversity, Gender Identity and LGBTIQ + Studies
Expert 5	University of the Basque Country	Research Consultant, Development and International Cooperation and Human Rights

**Table 5:** General and Personal Belief in a Just World Scale Scores.

Interview	GBJW	PBJW
1	15	26
2	18	18
3	20	18
4	10	15
5	23	25
6	19	24
7	8	12
8	14	24
9	14	21
10	16	22
11	25	28
12	22	28
13	18	22
14	21	25
15	20	27
